# Arachidonic Acid Metabolite 19(S)-HETE Induces Vasorelaxation and Platelet Inhibition by Activating Prostacyclin (IP) Receptor

**DOI:** 10.1371/journal.pone.0163633

**Published:** 2016-09-23

**Authors:** Sorin Tunaru, Ramesh Chennupati, Rolf M. Nüsing, Stefan Offermanns

**Affiliations:** 1 Max-Planck-Institute for Heart and Lung Research, Department of Pharmacology, Ludwigstr. 43, 61231 Bad Nauheim, Germany; 2 Institute for Clinical Pharmacology, J.W. Goethe University Frankfurt, Theodor-Stern-Kai 7, 60590 Frankfurt, Germany; 3 Medical Faculty, J.W. Goethe University Frankfurt, Theodor-Stern-Kai 7, 60590 Frankfurt, Germany; Universidade Federal do Rio de Janeiro, BRAZIL

## Abstract

19(S)-hydroxy-eicosatetraenoic acid (19(S)-HETE) belongs to a family of arachidonic acid metabolites produced by cytochrome P450 enzymes, which play critical roles in the regulation of cardiovascular, renal and pulmonary functions. Although it has been known for a long time that 19(S)-HETE has vascular effects, its mechanism of action has remained unclear. In this study we show that 19(S)-HETE induces cAMP accumulation in the human megakaryoblastic leukemia cell line MEG-01. This effect was concentration-dependent with an EC_50_ of 520 nM, insensitive to pharmacological inhibition of COX-1/2 and required the expression of the G-protein G_s_. Systematic siRNA-mediated knock-down of each G-protein coupled receptor (GPCR) expressed in MEG-01 followed by functional analysis identified the prostacyclin receptor (IP) as the mediator of the effects of 19(S)-HETE, and the heterologously expressed IP receptor was also activated by 19(S)-HETE in a concentration-dependent manner with an EC_50_ of 567 nM. Pretreatment of isolated murine platelets with 19(S)-HETE blocked thrombin-induced platelets aggregation, an effect not seen in platelets from mice lacking the IP receptor. Furthermore, 19(S)-HETE was able to relax mouse mesenteric artery- and thoracic aorta-derived vessel segments. While pharmacological inhibition of COX-1/2 enzymes had no effect on the vasodilatory activity of 19(S)-HETE these effects were not observed in vessels from mice lacking the IP receptor. These results identify a novel mechanism of action for the CYP450-dependent arachidonic acid metabolite 19(S)-HETE and point to the existence of a broader spectrum of naturally occurring prostanoid receptor agonists.

## Introduction

Arachidonic acid is metabolized by cyclooxygenase (COX)-1 and -2 enzymes to prostanoids which regulate a plethora of physiological functions and contribute to various pathological states [1]. Their effects are mediated by specific plasma membrane receptors belonging to the superfamily of G-protein coupled receptors (GPCRs) [2]. An alternative pathway for the metabolization of arachidonic acid is mediated by cytochrome P450 (CYP450) enzyme family members which can convert arachidonic acid into epoxy-eicosatrienoic acids (EETs) and hydroxy-eicosatetraenoic acids such as 19- and 20-HETE [3–6]. Under basal conditions, 19(S)-HETE is the most abundant HETE produced by the rabbit kidney, its secretion being enhanced by angiotensin II [7]. *In vitro* studies show that multiple isoforms of class 2 CYP450 enzymes, such as isozymes 2E1, 2U1 and 2J9, can metabolize arachidonic acid to 19- and 20-HETE [8–10].

Experiments performed in perfused kidney from rabbits indicated that 19(S/R)-HETEs and 20-HETE are COX-1/2-dependent dilators of rabbit renal vasculature [7, 11]. Additional studies, however, showed that 19(S/R)-HETEs and 20-HETE have pro-contractile effects on rat thoracic aortic rings, the latter being sensitive to COX-1/2 inhibition [12]. Nevertheless, it has also been described that 19(S)-HETE, but not its (R) isomer or arachidonic acid, functions as a stimulator of Na^+^ /K^+^-ATPase in a concentration-dependent manner, thereby contributing to increased volume absorption in the rabbit renal proximal straight tubes [13]. Although the general consensus appears to be that 19(S)-HETE has mainly vasodilatory effects, its biological role and the mechanism of action still remain unclear.

Here we show that 19(S)-HETE is a full orthosteric agonist of the prostacyclin (IP) receptor and, by activating IP, can induce inhibition of platelet activation as well as relaxation of vessels from different vascular beds.

## Materials and Methods

### Reagents

19(S)-HETE and all related HETEs, cicaprost, arachidonic acid, PGE_2_, PGD_2_, cloprostenol, U46619, PGD_2_, Cay10441, were from Cayman Chemicals. FR122047 and NS398 were from Tocris. Forskolin, thrombin and sodium nitroprusside (SNP) were from Sigma-Aldrich and indomethacin from Alfa Aesar. [^3^H]-Iloprost (20 Ci/mmol) was from American Radiolabeled Chemicals.

### Intracellular cAMP determination

MEG-01 or other indicated cell types, seeded in white walled, clear bottom 96-well plate were stimulated with test substances in Hanks’ balanced salt solution (HBSS) including 1.8 mM CaCl_2_ and 10 mM glucose for 15 min at 37°C. Intracellular cAMP concentrations were determined by using an homogenous time-resolved fluorescence (HTRF)-based cAMP assay (Cisbio) following manufacturer’s instructions.

In case of heterologously expressed receptors, intracellular cAMP levels were measured by coexpression of a plasmid encoding a cytosolic cAMP-sensitive bioluminescent probe (GloSensor^™^ cAMP Assay, Promega). Forty-eight hours after transfection of receptors and cAMP probe, cells were removed from the incubator and medium was changed to HBSS containing 1.8 mM CaCl_2_, 10 mM glucose and 2% (v/v) of GloSensor^™^ cAMP reagent. Two hours after of incubation in dark at room temperature, intracellular cAMP was estimated in the “kinetic” mode by continuously recording the light produced over indicated time following ligand stimulation. For “end-point” determination, cells were stimulated with indicated ligands, and light generated was measured 15 minutes later with an integration time of 1250 ms. In both cases, light was recorded by a 96-well plate reader (Flexstation 3, Molecular Devices) and results were evaluated by SoftMaxPro software (Molecular Devices).

### Cell transfection and determination of [Ca^2+^]_i_

For studies of heterologously expressed receptors, COS-1 cells were seeded in white walls-clear bottom 96-well plates and transfected with plasmid containing cDNA for a calcium-sensitive bioluminescent fusion protein between aequorin and GFP[14] and plasmids containing the indicated receptors cDNA or control (empty vector, mock) at a concentration of 50 ng/well by using FuGENE 6 reagent (Promega) following manufacturer’s instructions. Forty eight hours later, cells were loaded with 5 μM coelenterazine *h* (Invitrogen) in HBSS containing 1.8 mM CaCl_2_ and 10 mM glucose for 2h at 37°C. Measurements were performed by using a luminometric plate reader (Flexstation 3). The area under each calcium transient was calculated by using SoftMaxPro software and expressed as area under the curve (AUC).

### Radioligand binding assay

To measure the equilibrium binding of [15-^3^H]-iloprost (20 Ci/mmol; American Radiolabeled Chemicals, Inc.) to IP receptor, COS-1 cells were seeded in 24-well plates. Twenty-four hours later, they were transfected with a plasmid containing human IP receptor cDNA using Fugene 6 (Promega) transfection reagent, according to the manufacturer’s instructions. Two days later, cells were rinsed once with ice-cold binding buffer (PBS + 0.5% fatty-acid free BSA) and competition binding assays were carried out by incubating transfected cells in binding buffer containing 10 nM [^3^H]-iloprost and indicated concentrations of unlabeled substances for 90 min at +4°C. Binding was stopped by three washing steps with ice-cold binding buffer. Thereafter, cells were lysed in lysis buffer (0.1% Triton X-200, 2N NaOH) and transferred to vials containing scintillation fluid (Ultima-Gold; Perkin-Elmer). Radioactivity was measured by a scintillation counter (Hidex 300SL).

### siRNA transfection and screening

MEG-01 cells seeded in 96 well plates were reverse transfected with pools consisting of four separate siRNAs against the indicated GPCR mRNAs at a concentration of 5 ng for each siRNA. Seventy-two hours later, 19(S)-HETE-induced cAMP increase was determined and ratios between effects on cells transfected with pools targeting a particular GPCR and effects on cells transfected with scrambled siRNA were determined.

### RNA isolation and quantitative RT-PCR

RNA isolation and transcription from MEG-01 and HUVECs were performed by using the RNeasy kit (Qiagen) according to the manufacturer’s instructions. Quantitative RT-PCR was done by using primers designed with the Roche’s online tool reagents and a Universal Probe Library assay from Roche.

### Genetic mouse models

IP-receptor deficient mice have been described elsewhere [15]. The mice used for experiments in this study were offspring of a breeding colony kept at the Institute for Clinical Pharmacology of the J. W. Goethe University, Frankfurt am Main (Germany). Mice were group-housed in ventilated cages (Techniplast Greenline) containing nesting material. They were kept under 12-hour light/12-hour dark cycle, at controlled ambient temperature of 23°C, 55% relative humidity with free access to water and fed ad libitum. Mice were regularly screened to ensure that they were pathogen–free. The maintenance of the animals and the experimental procedures were performed in accordance with the German animal welfare legislation.

### Isometric tension measurements

2 mm-long aortic segments were mounted in a conventional myography set-up (610-M, Danish Myo Technology) and bathed in Krebs buffer (119 mM NaCl, 4.7 mM KCl, 2.5 mM CaCl_2_x2H_2_O, 1.17 mM MgSO_4_x7H_2_O, 20 mM NaHCO3, 1.18 mM KH_2_PO_4_, 0.027 mM EDTA, 10 mM glucose) aerated with carbogen and containing, where indicated, 10 μM indomethacin or NS398 (1 μM) and FR122047 (10 μM). After 30 minutes equilibration, the resting tension was adjusted to 10 mN and contraction was initiated by the addition of U46619 (1 μM). 2–3 minutes after U46619 stimulation, acetylcholine (10 μM) was added to assess the endothelium-dependent relaxation of the aortic segments. After that, aortic segments were washed three times with Krebs buffer and were allowed 30 minutes equilibration before addition of the indicated ligands (phenylephrine + 19(S)-HETE, cicaprost and isoproterenol).

### Platelets preparation from mouse and aggregation measurements

Blood was collected from the retro-orbital plexus in a tube containing 10% (v/v) ACD buffer (85 mM trisodium-citrate dihydrate, 66.6 mM citric acid monohydrate and 111 mM anhydrous D(+) glucose, pH 4.5). Platelet-rich plasma (PRP) was obtained by centrifuging blood at 150 x g for 10 min at room temperature. Collected PRP was centrifuged again at 350 x g for 10 min. The platelet pellet was washed twice in Tyrode's buffer (137 mM NaCl, 2 mM KCl, 12 mM/ NaHCO_3_, 0.3 mM NaH_2_PO_4_, 5.5 mM glucose, 5 mM Hepes, pH 7.3) containing 0.35% bovine serum albumin and finally resuspended at a density of 5 × 10^5^ platelet/μl in the same buffer in the presence of 0.02 units/ml ADP scavenger apyrase, a concentration sufficient to prevent desensitization of platelet ADP receptors during storage. Platelets were kept at 37°C throughout all experiments. To determine platelet aggregation, light transmission was measured using washed platelets adjusted to a platelet concentration of 3 × 10^5^ platelets/μl with Tyrode's buffer containing CaCl_2_ (1 mM). Transmission was recorded on a 4-channel aggregometer (APACT Laborgeräte und Analysensysteme, Hamburg, Germany).

### Statistical analysis

Statistical analyses of differences between two groups were performed by non-parametric, unpaired, 2-tailed Mann-Whitney test. A *P* value less than 0.05 was considered significant.

## Results

### 19(S)-HETE specifically induces cAMP increases in MEG-01 cells

**Fig 1:**
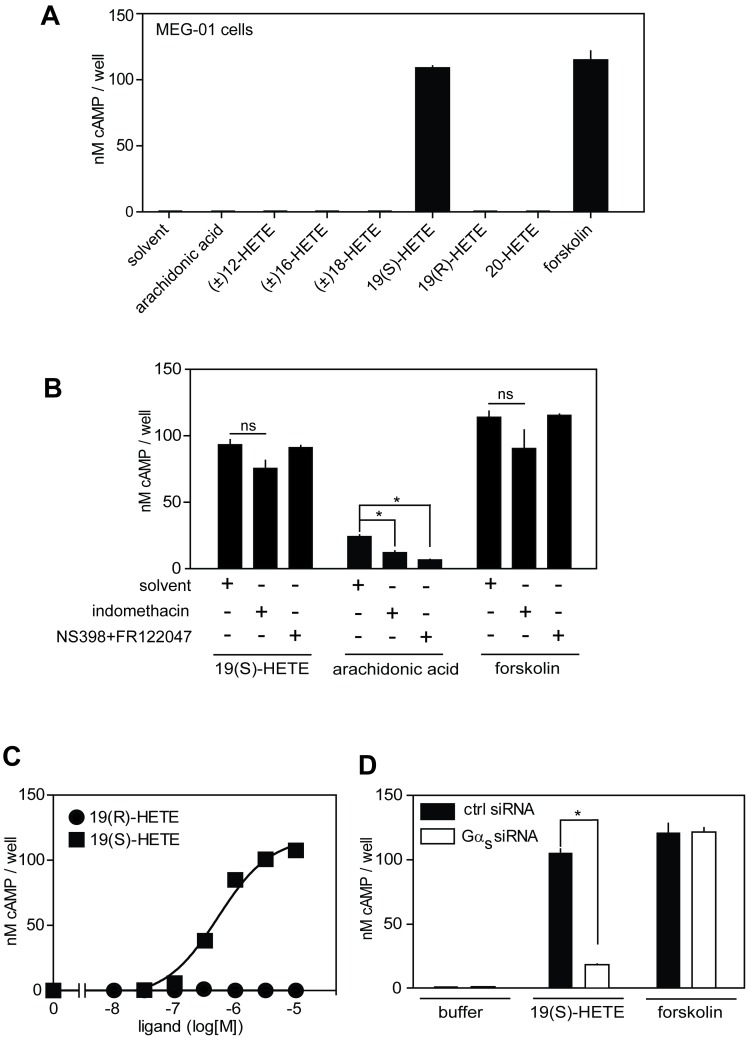
19(S)-HETE induces increase in cAMP levels in MEG-01 cells. (A) Effect of 19(S)-HETE and related compounds on intracellular cAMP levels in MEG-01 cells. cAMP concentration was measured 15 minutes after the addition of 1 μM of the indicated compounds and 10 μM of the positive control, forskolin. (B) Effects of COX-1/2 inhibition on 19(S)-HETE-induced cAMP increase in MEG-01 cells. Cells were pretreated for 30 minutes with indomethacin (10 μM), with NS398 (10 μM) and FR122047 (1 μM) or buffer (solvent) and were then stimulated with 19(S)-HETE (1 μM), arachidonic acid (3 mM) and forskolin (10 μM) for 15 minutes. (C) Effect of increasing concentration of 19(S)-HETE and its regiomer 19(R)-HETE on cAMP levels in MEG-01 cells. (D) Role of Gα_s_ in 19(S)-HETE- and forskolin-induced cAMP accumulation in MEG-01 cells. MEG-01 cells were reverse-transfected with scrambled siRNA (ctrl. siRNA) or Gα_s_ siRNA pools and 72h later were assayed for their responsiveness to 19(S)-HETE (1 μM) and forskolin (10 μM). Intracellular cAMP concentration was determined as described in Material and Methods. Shown are mean values ± SD, n ≥ 3. *, *P* ≤ 0.05; ns, not significant.

When studying hydroxylated arachidonic acid metabolites (HETEs) for specific cellular effects we identified 19(S)-HETE (19(S)-hydroxy-5Z,8Z,11Z,14Z-eicosatetraenoic acid), at a concentration of 1 μM, as an efficacious cAMP-elevating agent in cells of the megakaryoblastic leukemia cell line MEG-01 (Fig 1A). This stimulatory effect was dependent on the presence of the hydroxy-group because arachidonic acid, when tested at the same concentration, was inactive. Interestingly, none of the other related hydroxy-arachidonic acids, which differ by the location of the hydroxy group on the arachidonic acid backbone, were able to increase cAMP levels. Furthermore, the stereoisomer 19(R)-HETE was also inactive (Fig 1A), indicating that 19(S)-HETE-induced increase in the intracellular cAMP concentration in MEG-01 cells is a specific phenomenon.

Similar to arachidonic acid, HETEs can serve as substrates for intracellular COX-1/2 enzymes to produce prostanoids and/or hydroxy-prostanoids which may act as modulators of prostanoid receptors to initiate specific cellular signaling events [16]. In order to test whether 19(S)-HETE increases intracellular cAMP concentration in a COX-1/2-dependent manner, we examined the effect of pharmacological inhibition of COX-1/2 enzymes on the ability of 19(S)-HETE to increase cAMP levels in MEG-01 cells. Pretreatment of cells with the COX-1/2 inhibitor indomethacin at a maximally effective concentration of 10 μM [17, 18] as well as with a mixture of the specific COX-1 and COX-2 blockers FR122047 and NS398 [19, 20] did not affect the stimulatory effect of 19(S)-HETE (Fig 1B). In contrast, the increase in intracellular cAMP produced by the prostanoid precursor, arachidonic acid, given at a relatively high concentration of 3 mM was significantly reduced by pharmacological inhibition of COX-1/2 enzymes. Forskolin, which directly activates adenylyl cyclases [21] induced a cAMP accumulation which was not affected by COX-1/2 inhibitors (Fig 1B). The cellular effect of 19(S)-HETE was concentration-dependent and occurred with an EC_50_ of 520 nM whereas 19(R)-HETE remained inactive at concentrations up to 10 μM (Fig 1C). These data indicate that 19(S)-HETE is a potent and specific activator of a cellular signaling pathway which results in cAMP formation in a COX-1/2 independent manner.

To test whether the intracellular increase in cAMP concentration in response to 19(S)-HETE was mediated by a G_s_-coupled GPCR, we reverse transfected MEG-01 cells with a pool of four independent small interfering RNAs (siRNAs) targeting Gα_s_ mRNA. This resulted in a reduction of Gα_s_ mRNA by 80% (data not shown) as well as in a strong reduction of 19(S)-HETE-induced cAMP accumulation, whereas forskolin was still able to elevate intracellular cAMP levels similarly to control siRNA-transfected cells (Fig 1D). These data suggest that a GPCR coupled to G_s_ mediates the increase in cAMP levels after exposure of MEG-01 cells to 19(S)-HETE.

### 19(S)-HETE functions as an IP receptor agonist to increase cAMP in MEG-01 cells

**Fig 2:**
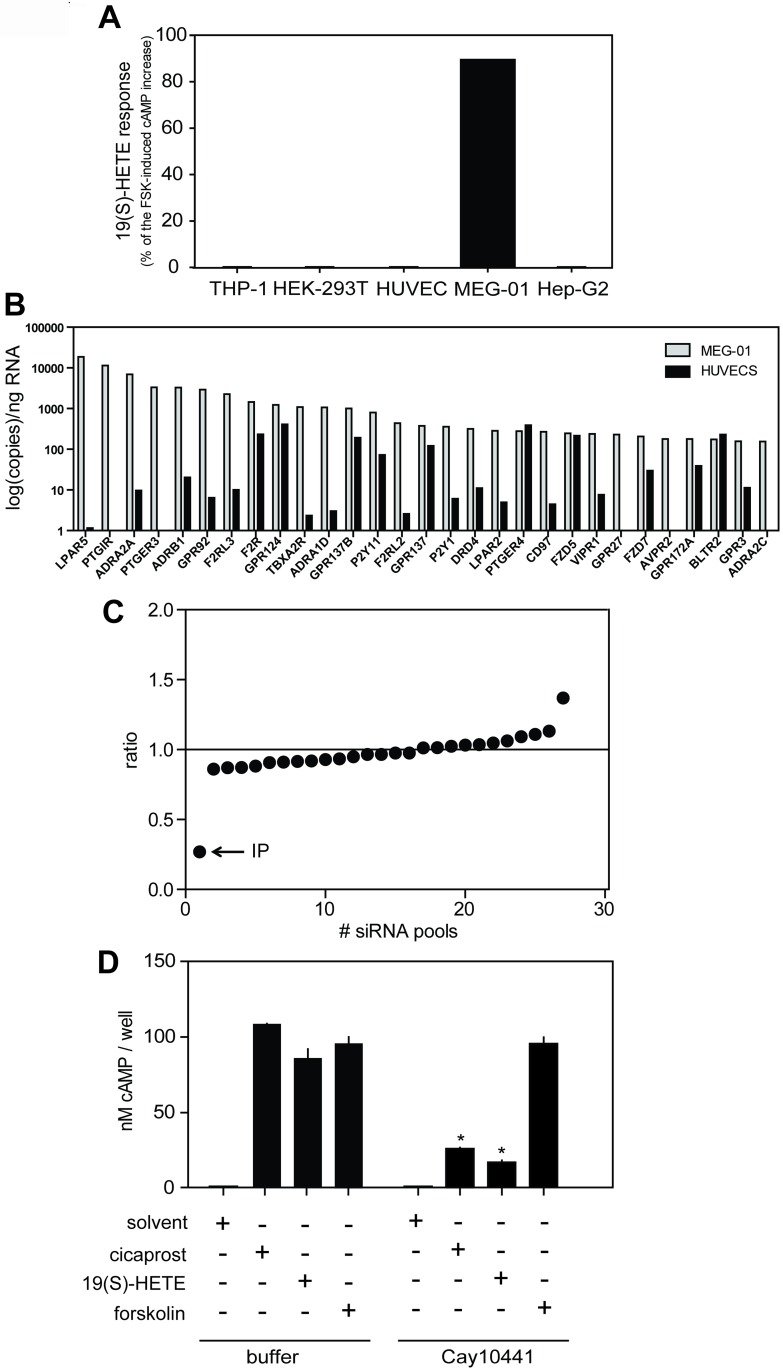
IP receptor mediates the effect of 19(S)-HETE in MEG-01 cells. (A) Effect of 1 μM 19(S)-HETE on cAMP levels in human monocytic cells (THP-1), human embryonic kidney cells (HEK-293T), human umbilical endothelial vein cells (HUVECs), human megakaryoblastic cells (MEG-01) and human liver hepatocellular carcinoma cells (Hep-G2). 19(S)-HETE response is shown as percentage of the forskolin (FSK)-induced cAMP increase. (B) Quantitative expression analysis of GPCRs in cells that respond (MEG-01) and in cells that do not respond (HUVECs) to 19(S)-HETE. (C) Ratio between effects induced by 19(S)-HETE (1 μM) in cells transfected with a pool of siRNA directed against a particular receptor and effects induced in cells treated with scrambled siRNA. Graph represents ranked averages of six independent experiments performed with 27 siRNA pools. (D) Effect of IP receptor antagonist, Cay10441 (3 μM), on 19(S)-HETE (1 μM) and cicaprost (1 μM)-induced cAMP accumulation in MEG-01 cells. Shown are mean values ± SD, n ≥ 3. *, *P* ≤ 0.05 (compared to buffer-treated controls).

Among several cells and cell lines studied, only MEG-01 cells were responsive to 19(S)-HETE (Fig 2A), indicating that a putative GPCR would only be expressed in MEG-01 cells. We therefore performed quantitative expression analysis of all non-sensory GPCRs in MEG-01as well as in other non-responsive cells such as human umbilical vein endothelial cells (HUVECs), in which 19(S)-HETE was inactive (Fig 2A and 2B). In order to identify a putative receptor responding to 19(S)-HETE, we reverse-transfected MEG-01 cells with pools of siRNAs targeting receptor mRNAs which showed significant expression in MEG-01 cells but not in HUVECs. As shown in the Fig 2C, siRNAs directed against the mRNA encoding the prostacyclin (IP) receptor strongly inhibited the increase in cAMP concentration induced by 19(S)-HETE. Furthermore, the IP receptor antagonist Cay104401 blocked the effects of 19(S)-HETE and of the IP-receptor agonist cicaprost in MEG-01 cells whereas forskolin-induced cAMP accumulation remained unaffected, strongly indicating that the IP receptor mediates 19(S)-HETE-induced elevation in cAMP levels (Fig 2D).

**Fig 3:**
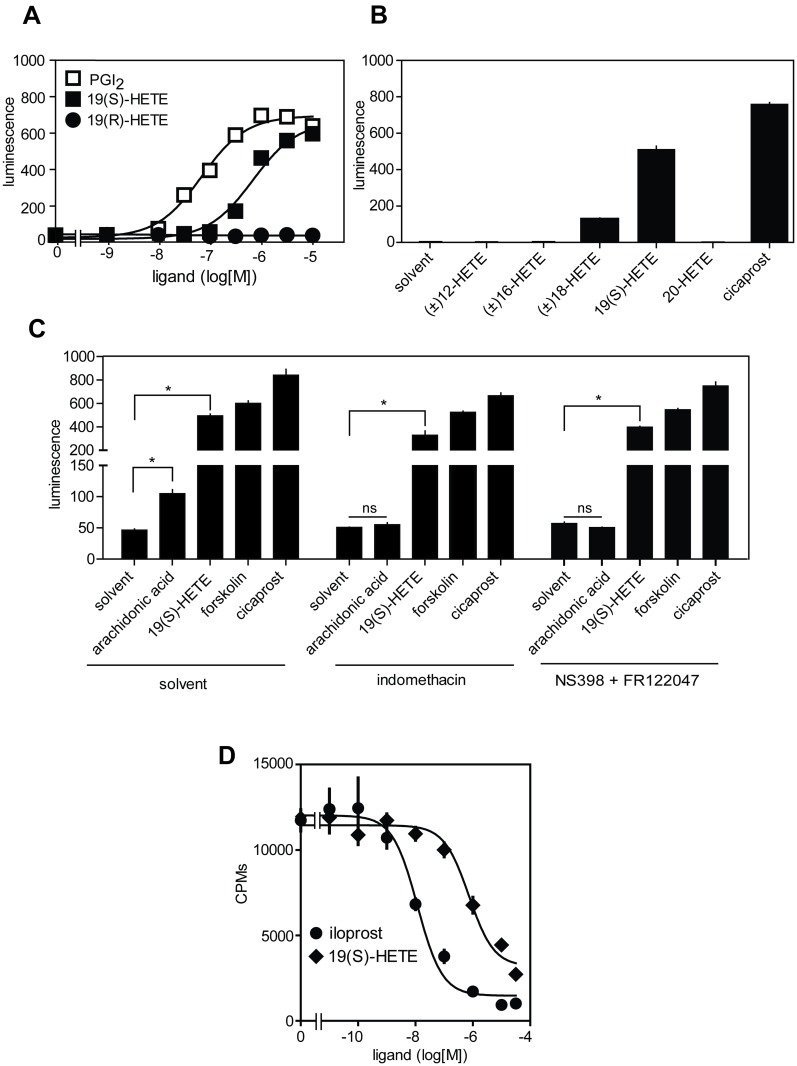
19(S)-HETE is an orthosteric prostacyclin receptor agonist. (A) Effect of increasing concentrations of PGI_2_, 19(S)-HETE and 19(R)-HETE on cAMP levels in COS-1 transfected with IP receptor and an intracellular cAMP-sensitive bioluminescent probe (see Material and Methods). (B) Effect of different HETEs at 1 μM and of cicaprost (1 μM) on cAMP levels in COS-1 expressing IP receptor and the intracellular bioluminescent cAMP probe. (C) Effect of COX-1/2 blockers indomethacin (10 μM), NS-398 (10 μM) and FR122047 (1 μM) on formation of cAMP induced by 19(S)-HETE (1 μM), arachidonic acid (3 mM), cicaprost (1 μM) and forskolin (10 μM) in COS-1 cells expressing IP receptor. (D) Effect of iloprost and 19(S)-HETE on binding of 10 nM [^3^H]-iloprost to IP receptor expressed in COS-1 cells. Shown are mean values ± SEM, n ≥ 3. *, *P* ≤ 0.05; ns, not significant.

To further test whether 19(S)-HETE functions as an IP receptor agonist, we heterologously expressed the human IP receptor in COS-1 cells together with an intracellular cAMP-sensitive bioluminescence probe (see Material and Methods). As shown in the Fig 3A, 19(S)- but not its stereoisomer 19(R)-HETE was able to concentration-dependently activate the IP-receptor with an EC_50_ of 567 nM, consistent with its potency determined in MEG-01 cells. On the other hand, cells expressing empty vector did not respond to prostacyclin or 19(S)-HETE at concentrations up to 10 μM (data not shown). Consistent with the observed effects in MEG-01 cells (Fig 1A) related HETEs did not activate the IP receptor (Fig 3B). Indomethacin and a mixture of the specific inhibitors of COX-1 and COX-2, FR122047 and NS-398, did not reduce IP-mediated cAMP increase after exposure of cells to 19(S)-HETE and cicaprost. In contrast, the arachidonic acid-induced cAMP increase was blocked by COX-1/2 inhibition, whereas forskolin effect remained unchanged (Fig 3C). In addition, 19(S)-HETE was able to displace ^3^H-iloprost from IP receptor expressed in COS-1 with an Ki of 660 nM (Fig 3D). Taken together, these data show that 19(S)-HETE is a specific orthosteric agonist of the IP receptor.

**Fig 4:**
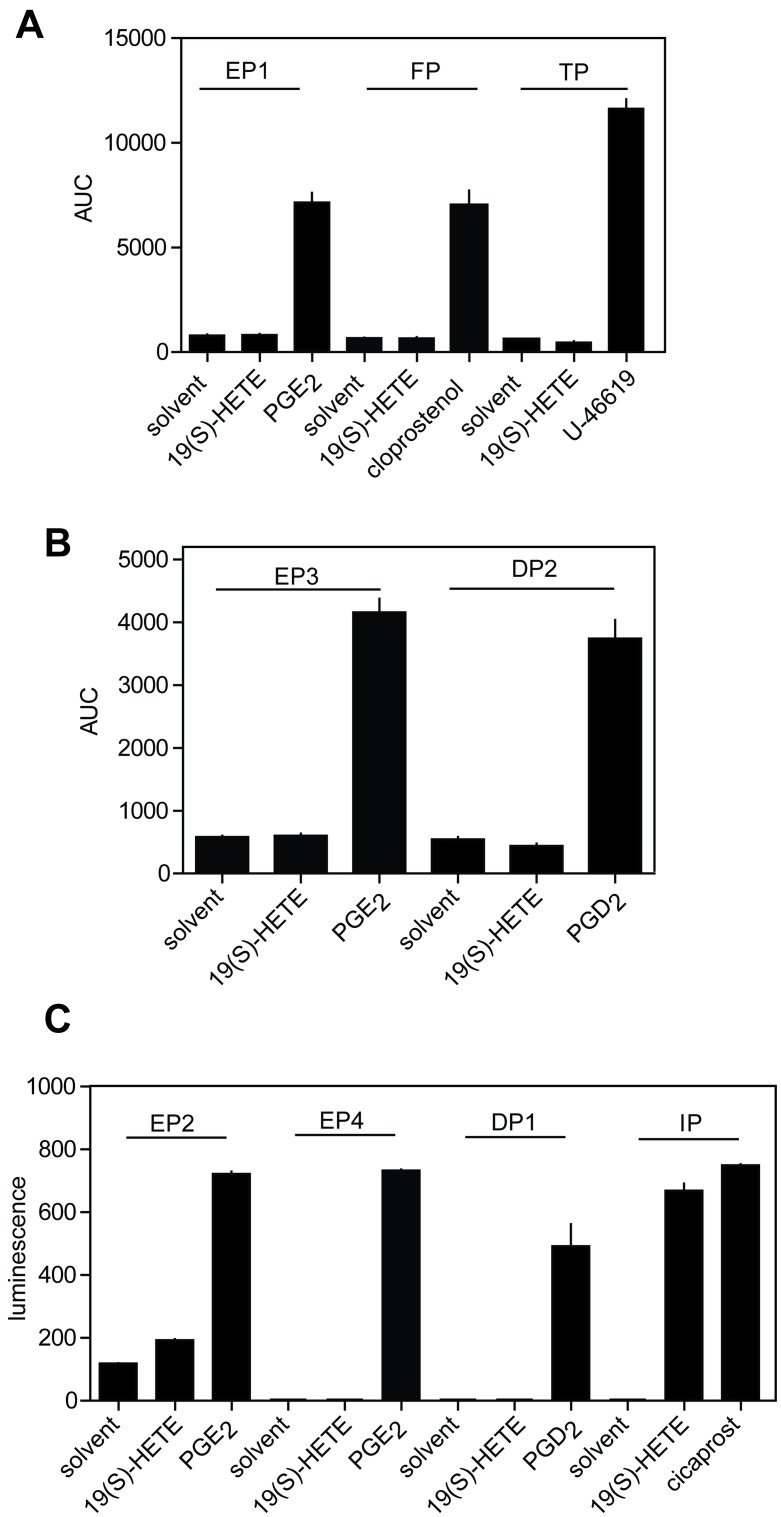
19(S)-HETE is a selective IP receptor agonist. (A) COS-1 cells expressing G_q_/G_11_-coupled prostanoid receptors together with a Ca^2+^-sensitive bioluminescent fusion protein were exposed to 19(S)-HETE (3 μM) and their cognate prostanoid receptors ligands at 3 μM. EP1, PGE_2_ receptor subtype 1; FP, prostaglandin F_2α_ receptor; TP, thromboxane A_2_ receptor. AUC, area under the curve of agonist-induced calcium transients recorded for 100 seconds. (B) Effect of 19(S)-HETE (3 μM) on various G_i_-coupled prostanoid receptors expressed together with the promiscuous G-protein α-subunit Gα_15_ in COS-1 cells using a Ca^2+^-sensitive bioluminescent probe. Functionality of receptors was verified by recording responses after stimulation with the specific prostanoid receptors at 3 μM. EP3, PGE_2_ receptor, subtype 3; DP2, prostaglandin D_2_ receptor. (C) G_s_-coupled prostanoid receptors were heterologously expressed together with a cAMP-sensitive bioluminescence probe and stimulated with 3 μM of 19(S)-HETE or their endogenous ligands. EP2 and EP4, prostaglandin E_2_ receptors, subtypes 2 and 4; DP1, prostaglandin D_1_ receptor; IP, prostacyclin receptor. Luminescence refers to the light generated 15 minutes after ligand stimulation and recorded with an integration time of 1250 ms.

To test whether 19(S)-HETE functions as a specific agonist of prostacyclin receptors we heterologously expressed other prostanoid receptors in COS-1 cells together with an intracellular bioluminescent-sensitive calcium probe [14], and a promiscuous G-protein alpha subunit Gα_15_ [22]. While all prostanoid receptors were activated by their respective ligands, none of them except the IP receptor responded to 19(S)-HETE (Fig 4A–4C).

### 19(S)-HETE relaxes arterial blood vessels and inhibits platelet activation through the IP receptor

**Fig 5:**
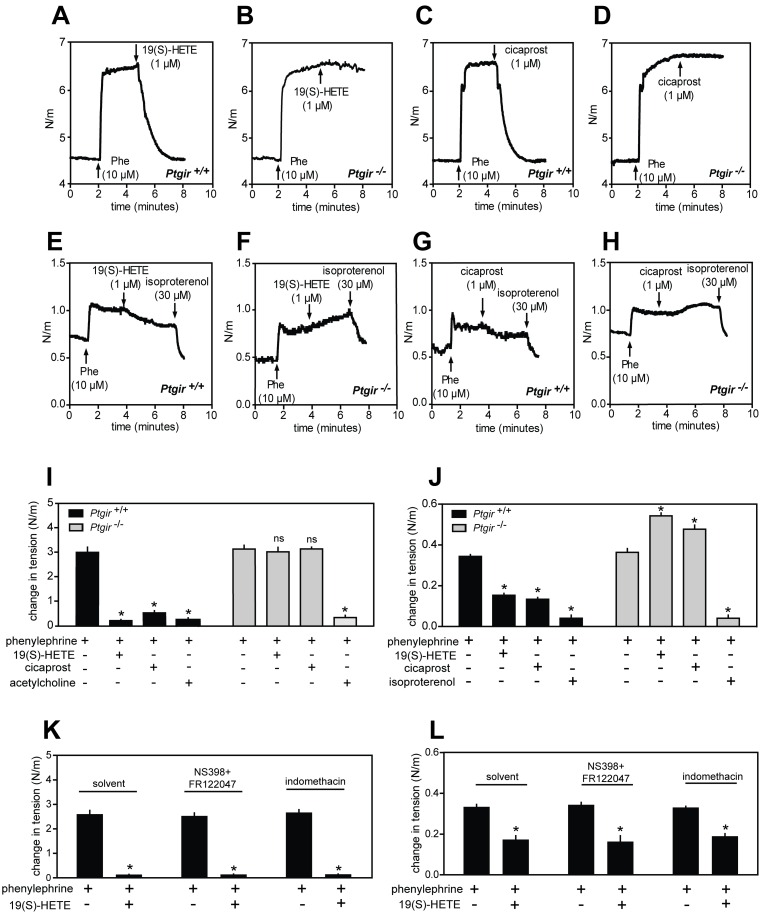
Vasorelaxant effects of 19(S)-HETE are mediated by the IP receptor. (A-D) Effects of 19(S)-HETE (1 μM) or cicaprost (1 μM) on isolated mouse mesenteric arterial segments pre-contracted with 10 μM phenylephrine from wild-type (A, C) or from IP receptor deficient mice (B, D). (E-H) Effects of 19(S)-HETE (1 μM), cicaprost (1 μM) and isoproterenol (30 μM) on aortic segments pre-contracted with phenylephrine (10 μM). (I, J) Quantification of agonist effects shown in panels A-D and E-H expressed as difference between agonist-induced aortic segment tension changes and basal levels. (K-L) Effect of NS398 (10 μM) and FR122047 (1 μM), or of indomethacin (10 μM) on the ability of 19(S)-HETE (1 μM) and cicaprost (1 μM) to induce relaxation of isolated mesenteric arteries (K) and aortic segments (L) pre-contracted by 10 μM phenylephrine. Shown are mean values ± SEM, n ≥ 3. *,*P* ≤ 0.05 (compared to contraction induced by phenylephrine alone).

Activation of IP receptor expressed in smooth muscle cells of vessels and subsequent cAMP generation causes vasorelaxation [15]. Because 19(S)-HETE was previously described to induce relaxation of renal arteries we analyzed whether similar effects can be observed in resistance arteries such as mesenteric arteries as well as in larger vessels such as murine thoracic aorta. Isometric tension recordings from mouse mesenteric artery segments mounted in a wire-myograph and bathed in a physiological buffer showed that 19(S)-HETE as well as cicaprost were able to strongly relax arterial segments pre-contracted by phenylephrine (Fig 5A, 5C and 5I). This effect was not affected by COX-1/2 inhibition with indomethacin or a mixture of NS398 and FR122047 (Fig 5K). In segments isolated from mice carrying a deleted *Ptgir* gene, which encodes the IP-receptor, 19(S)-HETE- and cicaprost-induced relaxations were completely abolished although their response to acetylcholine was not affected by IP-receptor deficiency (Fig 5B, 5D and 5I). Similarly, 19(S)-HETE and cicaprost also induced relaxation of phenylephrine pre-contracted aortic segments, although at a lower efficacy than in mesenteric segments (Fig 5E, 5G and 5J). Again, this effect was not influenced by COX-1/2 inhibition (Fig 5L) but it was abolished in vessels from mice lacking the IP receptor. Interestingly, in isolated aortic segments 19(S)-HETE and cicaprost significantly enhanced phenylephrine-induced contractions. (Fig 5F, 5H and 5J). These data show that 19(S)-HETE is able to elicit vasodilatory effects by directly activating the IP receptor.

**Fig 6:**
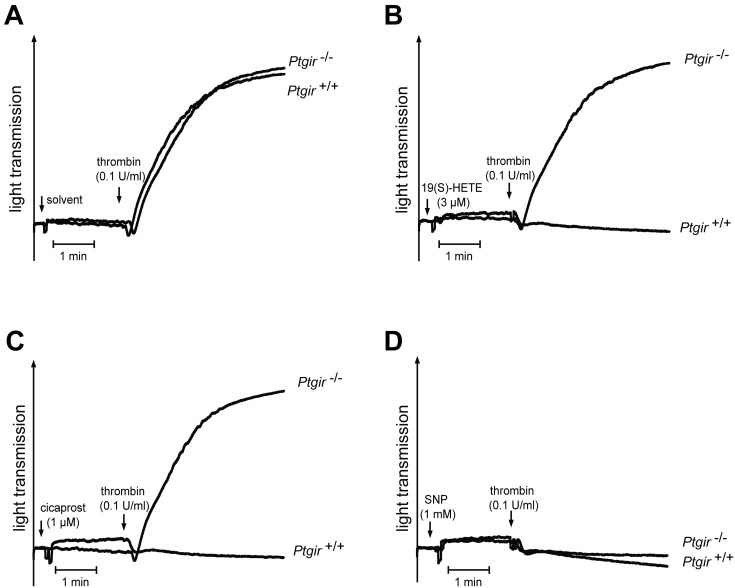
Platelet-inhibitory effects of 19(S)-HETE are mediated by IP receptor. (A-D) Effect of 19(S)-HETE (3 μM), cicaprost (1 μM) and sodium nitroprusside (SNP) (1 mM) pretreatment of platelets on aggregation of platelets isolated from wild-type (*Ptgir*^*+/+*^) and IP-receptor-deficient mice (*Ptgir*^*-/-*^) induced by 0.1 U/ml thrombin. Shown are representative aggregation traces of at least 3 independently performed experiments.

Since the IP receptor can also mediate platelet-inhibitory effects, we tested whether 19(S)-HETE is able to affect thrombin-induced platelets aggregation. As shown in Fig 6, pretreatment of platelets with 19(S)-HETE and cicaprost blocked the aggregatory responses of isolated mouse platelets to thrombin. In contrast, the platelet-inhibiting effects of 19(S)-HETE and cicaprost were absent in platelets from mice lacking IP receptor whereas inhibition of platelet function by the NO-donator sodium nitroprusside was not affected by the absence of IP receptor (Fig 6).

## Discussion

In this study we found that 19(S)-HETE is an efficacious cAMP elevating metabolite of arachidonic acid in MEG-01 cells by acting as an orthosteric agonist of IP receptor. Consistent with this, we could demonstrate that 19(S)-HETE has platelet-inhibiting and vasorelaxant effects which are mediated by the IP receptor. 19(S)-HETE is produced together with 20-HETE by several CYP450 isoforms [8, 10] and it is the major CYP450 metabolite of arachidonic acid released by the kidney under basal conditions as well as after injection of angiotensin II [23]. Several studies have analyzed the vascular effects of 19(S/R)-HETEs and the related 20-HETE. It has been reported that 20-HETE and, with several fold lower potency and efficacy, also 19(S)-HETE can induce contraction of isolated aortic segments from rat [12]. The effect of 20-HETE was shown to be sensitive to COX-1/2 inhibition by indomethacin [12]. Another study showed that 19(S)-HETE can reduce the perfusion pressure of isolated rabbit kidney, which was interpreted as a vasorelaxant effect. This effect was dependent on the functionality of COX-1/2, as it was abolished by indomethacin treatment [11]. Here we show that 19(S)-HETE has a direct vasorelaxant effect in isolated mesenteric artery and aorta segments from mice which was insensitive to pharmacological inhibition of COX-1/2. Furthermore, this vasorelaxant effect required the expression of IP receptor because in arteries and aorta segments isolated from mice lacking the *Ptgir* gene, this vasorelaxant effect was absent. Of note, 19(S)-HETE and cicaprost had a small procontractile effect on phenylephrine pre-contracted aortic rings. An explanation for this could be that cicaprost and 19(S)-HETE can activate unknown targets mediating contraction, an effect which in wild-type vessels is overcome by IP-mediated relaxation [24, 25].

While Escalante et al. [12] observed a small vasocontractile effect of 19-HETE on rat thoracic aorta rings, we did not observe any effects under basal conditions in rings from mouse aortae but we found a small yet significant vasodilatory effect when aortic rings from mouse aortae had been pre-contracted. We have currently no explanation for this difference, but we conclude that in both cases the effects are rather small and of questionable biological significance. In contrast, we found very strong vasodilatory effects of 19(S)-HETE which were IP receptor-mediated in mesenteric arteries. However, in contrast to Carroll et al [11], we did not observe any difference after pretreatment with COX-inhibitors. This difference may be due to different experimental settings. While Carroll et al. [11] determined the effect of 19(S)-HETE on the perfusion pressure of isolated kidneys, we directly measured effects of 19(S)-HETE on vascular tone using a wire myography system. The effect of 19(S)-HETE on organ perfusion may be only indirectly linked to its potential vasodilatory activity, and many other, indirect factors, which involve COX activity may modulate or mediate this effect.

It has been long known that CYP450-dependent metabolites of arachidonic acid, including EETs and HETEs, are not just metabolic by-products but are also important regulators of physiological and pathophysiological processes, depending on their site of production [4–6]. Due to their hydrophobicity, they can be bound to tissue lipids [23], but upon hormonal stimulation they can be easily released to act in an autocrine or paracrine manner. Although a lot of studies focused on describing their biological roles, the mechanisms of action of EETs and HETEs remain, in most cases, a matter of debate. The 12-lipoxygenase metabolite of arachidonic acid, 12(S)-HETE, was shown to function as an agonist of the orphan GPCR GPR31 and to increase the invasiveness of PC3M adenocarcinoma cells in a GPR31-dependent manner [26]; other studies described 12(S)-HETE as a vasorelaxant, which appears to act as an antagonist of the thromboxane-A_2_ receptor [27]. Our data, showing that 19(S)-HETE is an agonist of the prostacyclin IP receptor, identify another GPCR as a receptor activated by an hydroxy-eicosatetraenoic acid, and it is tempting to speculate that several other biologically active metabolites of arachidonic acid, for which no mechanism of action has so far been described, may act through known or still to be de-orphanized GPCRs.

The IP receptor/prostacyclin pair plays a critical role in the regulation of blood pressure, and IP receptor deficient mice develop salt-sensitive hypertension and cardiac fibrosis [28]. Since 19(S)-HETE appears to be the most abundant HETE released from kidney, at concentrations which are equal or higher than that of prostaglandins [23] and based on its agonistic activity at the prostacyclin receptor, it is not unlikely that 19(S)-HETE contributes to blood pressure regulation or regulation of local blood flow by complementing prostacyclin effects. There is clear evidence that 19(S)-HETE can be produced under different conditions and in different organs. However, future work has to identify the exact cellular source of 19(S)-HETE. Based on our observation that 19(S)-HETE acting through the IP receptor is a strong platelet inhibitor, it will also be interesting to further explore the relationship of localized 19(S)-HETE formation and its local platelet-inhibitory activity.

Taken together, we have identified 19(S)-HETE as a full orthosteric prostacyclin IP receptor agonist able to exert various biological effects through IP receptor activation. Future work will be required to further explore the physiological and pathophysiological relevance of 19(S)-HETE-dependent regulation of vascular and other functions.
